# 2128. *In Vitro* Activity of Ceftibuten-Avibactam and Comparator Agents Against Resistant Enterobacterales from UTIs Collected Globally as Part of the ATLAS Surveillance Program, 2022

**DOI:** 10.1093/ofid/ofad500.1751

**Published:** 2023-11-27

**Authors:** Meredith Hackel, Gregory Stone, Daniel F Sahm

**Affiliations:** IHMA, Schaumburg, Illinois; Pfizer, Inc., Groton, Connecticut; IHMA, Schaumburg, Illinois

## Abstract

**Background:**

Increasing resistance among agents commonly prescribed to treat urinary tract infections (UTIs) indicate that new orally bioavailable agents are needed. Ceftibuten (CTB) is an orally administered third-generation cephalosporin and is in early clinical development being combined with an oral prodrug of avibactam (AVI). AVI is a non-β-lactam inhibitor of Ambler class A β-lactamases, including ESBLs and KPCs, class C (AmpC) β-lactamases, and some class D (OXA-48) β-lactamases. Ceftibuten-avibactam prodrug (CBA) is in early clinical development as a potential oral treatment for complicated urinary tract infections. This study evaluated the *in vitro* activity of CBA and oral comparators against Enterobacterales from UTIs with drug-resistant phenotypes collected globally as part of the 2022 Antimicrobial Testing Leadership and Surveillance (ATLAS) program.

**Methods:**

4,265 non-duplicate clinical isolates from UTIs were collected in 2022 in 55 countries. Susceptibility testing with CBA tested at a fixed concentration of 4 µg/mL AVI and comparators was performed by CLSI broth microdilution and interpreted using CLSI 2023 breakpoints. Nonsusceptible (NS)/resistant (R) phenotypes were based on 2023 CLSI breakpoints. MDR was defined as R to ≥1 agent from ≥3 drug classes.

**Results:**

CBA was the most active compound tested against all Enterobacterales, with the MIC_90_ value decreasing from 32 µg/mL to 0.12 µg/mL. A total of 96% of all isolates were inhibited at a CBA MIC of ≤1 µg/mL. Percent susceptible of oral comparators ranged from 63.4% to 76.8%. CBA maintained activity against NS/R subsets of Enterobacterales (MIC_90_ range, 0.25 to 4 µg/mL; 85.0% to 93.2% inhibited at ≤1 µg/mL), including MDR isolates (MIC_90_, >64 µg/mL; 81.7% inhibited at ≤1 µg/mL).

**Figure:**
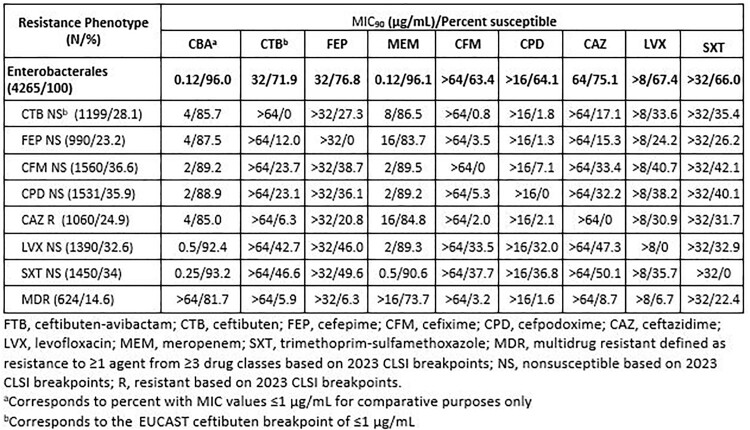

**Conclusion:**

Ceftibuten-avibactam demonstrated potent *in vitro* activity against multidrug-resistant Enterobacterales collected globally. Ceftibuten -avibactam prodrug could be an effective oral therapy for difficult-to-treat infections caused by multidrug-resistant Enterobacterales.

**Disclosures:**

**Meredith Hackel, PhD**, Pfizer Inc.: Honoraria|Venatorx: Paid fees for conducting the study and abstract preparation **Gregory Stone, PhD**, Pfizer: Stocks/Bonds **Daniel F. Sahm, PhD**, Merck & Co., Inc.: Honoraria|Pfizer Inc.: Honoraria|Venatorx: Paid fees for conducting the study and abstract preparation

